# Study on Nutritional Knowledge, Attitude and Behavior of Chinese School Football Players

**DOI:** 10.3390/children9121910

**Published:** 2022-12-06

**Authors:** Yao Chen, Yingshuang Sun, Zhiyun Liu, Donglin Hu

**Affiliations:** 1 School of Physical Education and Educational Science, Tianjin University of Sport, Tianjin 301617, China; 2 College of Physical Education, Hengyang Normal University, Hengyang 421008, China; 3 Department of Graduate Studies, Shenyang Sport University, Shenyang 110115, China; 4 Department of Physical Education, Nanjing Agricultural University, Nanjing 210095, China

**Keywords:** youth, dietary behavior, nutritional knowledge, football

## Abstract

Objective: This study aims to validate previous structural models of factors influencing dietary behavior changes and construct the knowledge, attitude, and behavioral models of youth school football players. Methods: 279 school football players aged 12–17 years in Grades 7–12 in Hunan Province, China, completed a questionnaire to collect data on sports nutrition knowledge, attitudes, and behavior. A structural equation model (SEM) was built based on the knowledge-attitude-behavior (KAB) model and the theory of planned behavior (TPB) model to introduce nutritional knowledge directly or by altering attitudes into the dietary behavior path of players. Four factors affecting dietary behaviors were involved in the hypothetical structure, which consists of the following hypotheses: (1) nutrition knowledge affects the attitude towards sports nutrition (H1); (2) nutrition knowledge affects dietary behaviors (H2); (3) nutrition knowledge affects subjective norms (H3); (4) nutrition knowledge affects perceived behavioral control (H4); (5) subjective norms affect dietary behaviors (H5); and 6) perceived behavioral control affects dietary behaviors (H6). Results: Confirmatory factor analysis (CFA) revealed that the reliability, convergent validity, and discriminant validity of the built SEM conformed to the measured relationships in each dimension. In the final structural model, it was found that nutrition knowledge had a direct impact on the attitudes of players and affected their dietary behaviors in a direct manner or through their subjective norms and perceived behavioral control. Conclusions: The results are in agreement with the TPB-based KAB chain and support the KAB theory for youth school football players in Hunan Province, China.

## 1. Introduction

In August 2020, the Ministry of Education of China and the General Administration of Sport of the People’s Republic of China jointly published the policy of “Opinions on Deepening the Integration of Sports and Education and Promoting the Healthy Development of Teenagers” [1]. From the policy level to promote school and competitive sports from surface, to depth of fusion, to practice [2,3,4]. As the first exploration project and basic project of educating people in school sports reform, “Campus Football” takes the lead in responding to the policy of integration of sports and education [5,6]. In the same year, the Ministry of Education of China published a “Notice of Action Plan for the Construction of the Eight Major Systems of National Youth Campus Football” [7]. The action plan establishes a working target of more than 30 million primary, middle, and high school students participating in football regularly by 2022. At present, China has completed the five-sphere integrated pattern of national school football promotion encompassing “specialist schools + collegiate high-level football teams + pilot counties (districts) + reform pilot regions + all-star training camps” [8]. The project covers 2038 schools, 40 pilot regions, five pilot counties (districts), and 28 all-star training camps [1]. As a result, campus football activities are gaining massive popularity throughout China. Consequently, a growing number of adolescents are participating in long-term systematic campus football training, showing comparative playing qualities to the youth team players in professional football clubs. Moreover, football is a sport with high requirements for physical fitness. It shows repeated changes in exercise intensity, from walking to running and running in a confrontational state [9]. The energy and nutrition needs of adolescent sports trainees are usually high because they increase their physical activity and physical growth [10,11]. Therefore, when the intake of energy and nutrients is insufficient, it will lead to poor sports performance and additional health problems. [11]. They should avoid getting sick and injured in training and competition, and the daily eating habits and eating behavior patterns on campus should be reasonable [12]. However, many factors (cultural beliefs, taste, food preference, convenience, availability, appetite, and attitude towards nutrition, as well as nutrition knowledge) hinder campus athletes from forming appropriate eating behaviors [13,14,15]. If campus football players want to make their daily eating habits reasonable, they need to make the corresponding behavior changes. This kind of change refers to the motivation, will, and course of action of giving up the eating habits that are harmful to health and adopting and maintaining healthy eating habits instead. The process is affected by many internal factors, such as knowledge, attitudes, intention, and stress and external factors such as social support and environment [16,17,18]. Many behavioral and social science theories and models have been used when trying to understand and enhance health behaviors [19,20,21,22]. Most of these theories exclude the influence of the environment on health behavior [23]. Nevertheless, to explore the change of behavior intention from the internal factors, the knowledge-attitude-behavior model (KAB model) on behavior change describes the order of knowledge, attitude, and behavior changes [23]. The KAB model has been used extensively in general health and nutrition education [23]. It suggests that knowledge guides attitude and affective evaluation of the topic, influencing behavior.

On the basis of previous studies, through a set of new data and confirmatory factor analysis, this study determined the factor structure related to the healthy eating behavior of campus football players. Another purpose is to determine the KAB model, that is, the relationship among campus football athletes’ nutritional knowledge, attitude, and behaviors. The hypothesis of the study is that acquiring nutrition knowledge can lead to changes in attitudes related to nutrition and diet, thus contributing to the improvement of behaviors.

## 2. Materials and Methods

### 2.1. Participants

The study used a two-step recruitment process. First, we selected schools and coaches through the Hunan Campus Football Management Office. In addition to being recognized as schools with campus football characteristics, these schools also participate in national football matches on behalf of Hunan Province because their football teams are excellent. Secondly, the campus football players were recruited through coaches (208 student players participated in the football training organized by the school). Therefore, this study adopts a non-probabilistic sampling method. Demographics of these students: There were 279 school football players in grades 7–12 (12–19 years old), 62.4% of whom were male (n = 174) and 37.6% of whom were female (n = 105).

This study was approved by the Sports Ethics Committee of Tianjin Institute of Physical Education (TJUS2022-027). After the cooperation with organizations (teenager sports clubs providing football training services for schools,, junior high schools, and senior high schools) was obtained, the participants were informed of the research purposes and procedures in both verbal and written forms. None refused to participate. All participants signed the informed consent form before the start of the research.

### 2.2. Tools

In order to measure the subjects’ sports nutrition knowledge, the “Sport Nutrition Knowledge Questionnaire (SNKQ)” [24] designed by Cheryl Jia Hui Teo et al. was used, in which the food items in some questions were adjusted according to the “Dietary Guidelines for Chinese Residents” and the Chinese diet environment [25]. Ultimately, the SNKQ contained 52 items, 66 questions, and 5 themes, with a total score of 66. The scoring method was as follows: 0 points for “Uncertain/Unknown,” 1 point for a correct answer, and −1 point for a wrong answer. The items in each theme section were scored separately, and all the items consisted of the whole SNKQ. The total score of each theme section was as below: Food (36), Micronutrient (6), Hydration (7), Exercise nutritional knowledge (10), and dietary replenisher (7). A higher score for a theme represents better mastery over the nutritional knowledge. In the end, the five themes were ranked according to their total scores.

In terms of assessing the dietary behavior of student athletes, the Eating Behaviour Questionnaire (EBQ) [24] (with a total score of 46), designed according to the theme classification in the SNKQ, was employed as the scoring system to quantify the dietary behavior of the participants. The student athletes’ diet recall for nearly three days was evaluated. Training sports nutritionists will conduct dietary assessments using food models and props such as bowls and measuring cups. The three-day food recall will be analyzed and scored accordingly. The regional food ingredients involved in the questions were adjusted according to the Chinese diet environment. In the EBQ, there were five types of themed dietary behaviors, and the higher the score for a theme, the better the dietary performance in that regard. The five topics include: Food groups (maximum 10), General Dictionary Habits (maximum 12), Hydration (maximum 4), Sports Nutrition specific Practices (maximum 12), and Supplements (maximum 8). In the end, the five types of dietary behaviors were ranked according to their scores.

Attitude has been used as a proxy measure of behavior since it was found to be systematically related to actions, suggesting that KAB relationships are correlative [26]. The Athlete’s Perspective on Nutrition instrument (APN) was developed to examine an athlete’s perspective toward dietary behavior for improving training and performance through nutrition, using the theory of planned behavior (TPB; Ajzen, 1991) as its primary framework [24]. In the scale, the theory of planned behavior (TPB) [27] was employed as its theoretical framework because the TPB has a similar theoretical attitude-behavior relationship to that in the KAB model. The TPB argues that behavioral results are affected by three factors: (1) an individual’s attitude toward the behavior, whether their opinion of the behavior in question is favorable or not; (2) subjective norms, including peer/social pressure, toward executing or not executing the behavior; and (3) the ease or difficulty of performing the behavior, termed perceived behavioral control [27]. In view of this, the “Attitude” in the KAB model is expanded into the “Student’s Perspective on Nutrition” using the theory of planned behavior as its primary framework [27,28]. The items of topics were defined according to the observable behavior (i.e., target, action, context, and time), and items were modified according to TPB questionnaire construction guidelines [27]. For each of these factors, there were 4 to 5 relevant items, which were answered via the 5-Point Likert Scale. The answers were scored from 1 (Strongly Disagree) to 5 (Strongly Agree). The higher the score, the more positive the evaluation of the item.

### 2.3. Survey Process

The survey subjects were from 8 specialist football schools recommended by the Hunan Provincial campus football authorities. All questionnaires were filled in online via a mobile phone under the guidance and supervision of the coaches so that discussions and information exchanges between the participants were avoided. Moreover, participants were not allowed to submit the questionnaires before all the questions were answered so as to ensure information completeness.

The questionnaires taken for an unduly long or short time were excluded to ensure the accurate and reliable information collected. The participants were recruited in May 2022, and the data collection was carried out in July 2022.

### 2.4. Statistics Analysis

IBM SPSS Statistics 26 was employed to analyze the data collected statistically, and the Kolmogorov-Smirnov (K-S) test was used to determine the normality of variables.

Confirmatory factor analysis (CFA) offers a measurement model based on structural equation modeling (SEM), which is used to pre-assume essential factors [29]. Its primary function is to determine latent structural factor information that cannot be directly measured [30]. The evaluation of CFA allows us to confirm whether or not the factor structure selected by testing the correlation among knowledge, attitude, and behavior is in agreement with the data collected or whether or not it should be modified. By constructing a structural equation model with the maximum likelihood estimation [31], the following hypotheses are proposed: (1) nutrition knowledge affects players’ attitudes towards sports nutrition (H1); (2) nutrition knowledge affects players’ dietary behaviors (H2); (3) nutrition knowledge affects players’ subjective norms (H3); (4) nutrition knowledge affects players’ perceived behavioral control (H4); (5) players’ subjective norms affect their dietary behaviors (H5); and (6) players’ perceived behavioral control affects their dietary behaviors (H6).

## 3. Results

### 3.1. Confirmatory Factor Analysis

CFA is used to investigate convergent validity, discrimination validity, and common method deviation (CMV) [30]. First, the basic information of data, such as the number of factors and sample size, should be understood. Then, if the sample size is less than five times the number of analysis items, it is recommended to increase the sample size.

It can be seen from Table 1 that five factors and 23 analysis items are used for CFA. The number of effective samples is 279, exceeding ten times the number of analysis items. Hence, the sample size is moderate.

Factor loading shows the correlation between the factor (latent variable) and the variable (manifest variable/measurement item) [30]. Generally, standard factor loading is employed to present the correlation between the factor and the variable (measurement item). A variable is considered significant when the standard factor loading exceeds 0.7, which means that the correlation is adequate. On the other hand, if the standard factor loading is low (e.g., below 0.4), the correlation between the variable and the factor is weak, and the variable may be removed [29,30]. As can be seen from Table 2, among the result variables, only the measured items 2 (B2) and 5 (B5) have standard loading less than 0.7 but greater than 0.6. Therefore, although there is no strong correlation, they have a good measured relationship.

Average variance extract (AVE) and combination reliability (CR) is used for the analysis of convergent validity. Under normal circumstances, if the AVE value is more significant than 0.5 and the CR value exceeds 0.7, it indicates high convergent validity [30]. However, if the value of AVE or CR is low, the factor may be removed to reanalyze the convergent validity. The calculation formulas are as below: AVE = Average (loading squared and then summed), CR = Sum(loading)^2 / [sum(loading)^2 + sum(e)], in which loading is the standard loading coefficient, and e is the residual standard loading coefficient [30,32]. The model is analyzed through CFA. As can be seen from Table 3, for the five factors, all the AVE values are more significant than 0.5, and all the CR values are greater than 0.7, which means that the data collected exhibited good convergent validity [30].

CFA can be used for discriminant validity research [33]. In Table 4, the values in the diagonal line are the AVE RMS values, and the rest are correlation coefficients. The AVE RMS value represents the “convergence” of the factor, whereas the correlation coefficient represents the correlation. As shown in Table 4, the AVE RMS values of sports nutrition knowledge, attitude, perceived behavioral control, subjective norms, and dietary behavior are 0.801, 0.766, 0.759, 0.747, and 0.720, respectively, all exceeding their corresponding maximum absolute value of the correlation coefficient (i.e., 0.419, 0.389, 0.415, 0.407, and 0.419). This suggests that there is satisfactory discriminant validity.

**Figure 1:**
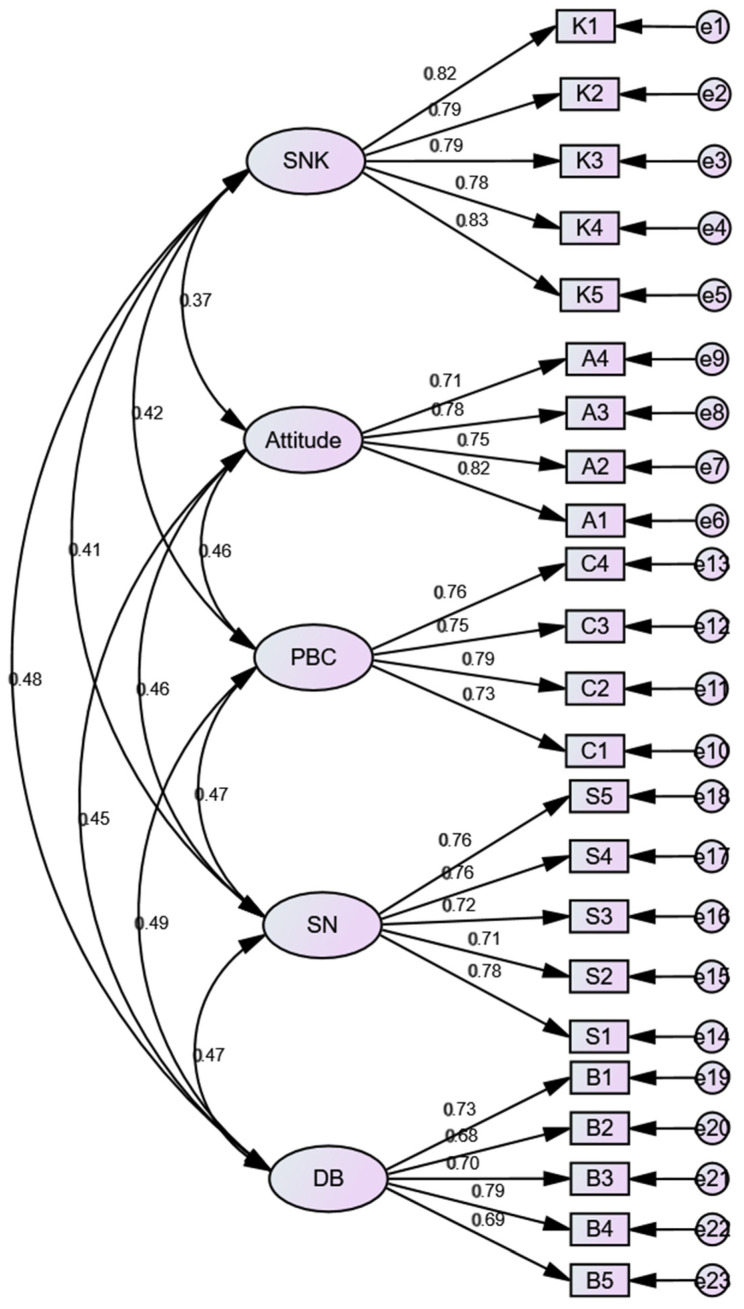
Standardized estimation of knowledge, attitude, and behavior CFA.

Model fit indices are used to analyze the overall fit validity of a model [34]. There are a number of fit indices, so it is not easy to make sure that all of them reach the standard in most cases. Hence, it is recommended to select a few commonly-used ones, such as CMIN/DF, GFI, RMSEA, RMR, CFI, NFI, and NNFI [34]. From Table 5, it can be observed that all the model fit indices are up to the standard, indicating that the degree of mode fit in Figure 1 is satisfactory.

### 3.2. Structural Equation Model

The SEM regression relationship table contains two relationships, namely the structural relationship and measured relationship. In both relationships, the standardized path coefficient value is typically used to express closeness in a relationship [34]. If the significance is exhibited, it means there is a significant structural/measured relationship; otherwise, there is no significant structural/measured relationship. If multiple path coefficients are found to have no significance, it means that the model is poor, and resetting the relationships in the model, i.e., adjusting the model, is recommended [32,34]. The first item in the measured relationship is the control item, so no z-value or *p*-value is output. As shown in Table 6, for the model, the regression coefficients of the latent variables relative to observed variables all are more significant than 0.5, indicating that the observed variable validity of the latent variables is good.

Model fit indices are used to analyze the overall fit validity of a model [31]. There are a number of fit indices, so it is not easy to make sure that all of them reach the standard. It can be seen from Table 7 that the indices, including CMIN/DF, GFI, RMSEA, CFI, NFI, and NNFI, all are up to the judgment standard, which suggests that the assumption model exhibits a satisfactory degree of fit. A default model refers to the initial value estimation indicator of the fitting model, which generally is less significant [31].

## 4. Discussion

This study aimed to identify the correlation among the sports nutrition knowledge, attitudes, and behaviors of the 12–17-year-old football players in Hunan Province and to provide empirical support for the KAB model. The results showed that sports nutrition knowledge was significantly positively correlated with attitudes, i.e., rich sports nutrition knowledge helped the players develop a positive attitude toward sports nutrition. The attitude toward sports nutrition was found to correlate positively with the behavior, and a positive attitude toward diet could predict sports nutrition behavior to a certain extent. Sports nutrition knowledge also showed a significant positive correlation with dietary behavior, which, to a certain extent, is similar to that in the KAB model.

In previous theoretical models concerning the correlation among knowledge, attitude, and behavior, knowledge has been identified as an external factor affecting the attitude toward behavior and the behavior itself [21,28,35]. The KAB model’s essential elements—improving knowledge, fostering motivation via beliefs and attitude, and facilitating action—are thought to have an impact on nutrition education’s success [36]. The KAB model involves behavior change cognitive models in the theories of social learning and rational behavior that concern society, psychology, and behavior [21,22,37]. All these models include the causal inference in perception, attitude, and behavior. Nonetheless, human behavior changes are so complicated—involving many internal and external factors affecting behaviors—that none of the above theoretical models is potent enough to describe them as a whole [27,28]. Most theories do not clearly explain how to measure and operate some intervention behaviors and influencing factors, but it is suggested that researchers develop measurement standards that are suitable for their own culture [16]. SNKQ integrates the latest development in sports nutrition and contextualizes the Chinese campus football players. When considering the direction of variable relations in the final model, Lee Herschberger’s rule is followed: the direction of all links is based on theory and previous research. The relationship direction in the knowledge attitude behavior chain is based on KAB theory. According to most theoretical models of the relationship between knowledge, attitude, and behavior, knowledge is the background factor that affects the attitude towards behavior and behavior itself [16,17,21].

The significant correlation between nutrition knowledge and attitude in Cheryl Jia Hui Teo’s research also supports this model [24]. This research believes that knowledge is related to the TPB theory’s attitude, subjective norms, and perceived behavior control [14]. This is also one of the bases for the hypothesis of this study.

**Figure 2:**
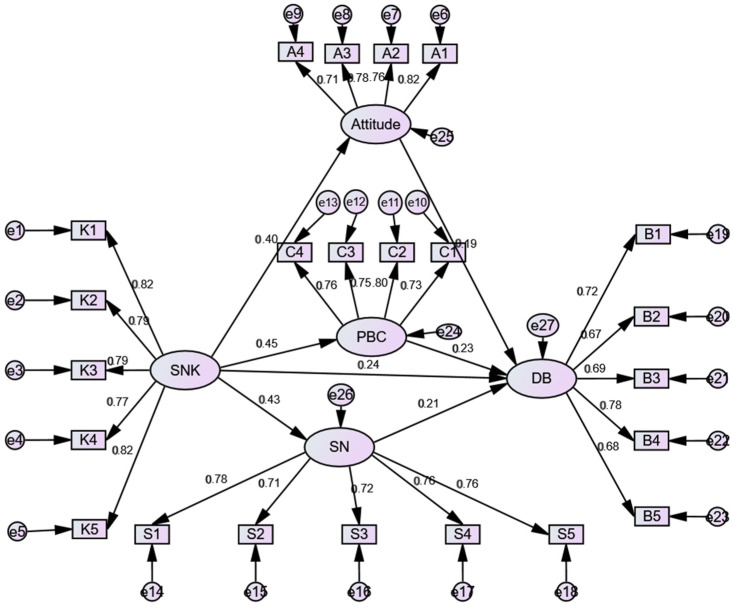
Standardized estimation of the correlation among knowledge, attitude, and behavior based on the final SEM model.

It can be seen from Figure 2 that the results showed that the path coefficient value of athletes’ sports nutrition knowledge and sports nutrition attitude was 0.4 > 0 (*p* < 0.01). The path coefficient value of the influence of sports nutrition knowledge on athletes’ sports nutrition behavior was 0.236 > 0 (*p* < 0.01), which shows that athletes’ sports nutrition knowledge can have a positive effect on athletes’ sports nutrition behavior; the path coefficient between sports nutrition knowledge and athletes’ subjective norm was 0.434 > 0 (*p* < 0.01), which indicated that sports nutrition knowledge could positively affect athletes’ subjective norm; and the path coefficient of sports nutrition knowledge and athletes’ perceived behavior control was 0.451 > 0 (*p* < 0.01), indicating that sports nutrition knowledge could positively affect athletes’ perceived behavior control. Furthermore, the path coefficient of athletes’ subjective norm and sports nutrition behavior is 0.209 > 0 (*p* < 0.01), which indicates that the perceived behavior control of sports can positively affect athletes’ sports nutrition behavior. Finally, the path coefficient of sports perception behavior control and sports nutrition behavior of athletes is 0.231 > 0 (*p* < 0.01), which indicates that sports perception behavior control can positively affect the sports nutrition behavior of athletes.

These results all confirm the theoretical basis of dietary behavior changes in youth football players, thereby supporting the causal chain theory concerning the sports nutrition knowledge, attitude, and behavior of youth school football players. Hence, the hypotheses mentioned above are supported:

(1) Sports nutrition knowledge can affect the attitude toward sports nutrition (H1).
(2) Sports nutrition knowledge can affect dietary behavior (H2).
(3) Sports nutrition knowledge can affect subjective norms (H3).
(4) Sports nutrition knowledge can affect perceived behavioral control (H4).
(5) Subjective norms can affect dietary behavior (H5).
(6) Perceived behavioral control could affect dietary behavior (H6).

This study has several limitations. First, there is no design to set up a control group in the same school to observe the statistically significant difference of variables. Second, since the accuracy of reported energy intake cannot be determined due to the lack of accurate collection of information on the physical activity level, the diet self-assessment mobile app and sportswear equipment can be used to make up for this in subsequent studies. Third, the study did not take the exercise nutrition curriculum as an intervention condition to observe the differences in exercise nutrition eating behavior of different subjects.

## 5. Conclusions

According to the survey results of SNKQ, APN, and EBQ, we can understand the sports nutrition knowledge, athletes’ attitudes towards healthy eating behavior, and the athletes’ healthy eating behavior level of campus football players in Hunan Province. Combining KAB and TPB theoretical models, this paper explored the relationship between sports nutrition knowledge, attitude, and sports eating behavior of student athletes through confirmatory factor analysis.

The results suggested that coaches consciously integrate sports nutrition knowledge into the training plan while making daily football training plans. In addition to incorporating sports nutrition knowledge into athlete training plans, coaches should also teach students to access diet and nutrition information on the Internet (for example, the diet and nutrition management mobile app). In addition, on the home school exchange day, the coach should communicate sports nutrition information with the student’s parents. At the level of policy management of campus football projects, the Ministry of Education needs to add sports nutrition to the training courses of campus football coaches. Only in this way can excellent young football players be cultivated under the sports-education integration policy.

## Figures and Tables

**Table 1:** CFA basic summary.

Factor	Num
Sport Nutrition Knowledge	5
Attitude	4
Perceived behavioral control	4
Subjective norms	5
Dietary behavior	5
Total	23
Effective samples	279

Note: Summary of quantity of measurement items corresponding to each factor. Effective samples: The effective sample size is 279, 10 times more than the total number of analysis items (23).

**Table 2:** Factor loading coefficients.

Factor	Manifest Variable	Coef.	Std. Error	CR	*p*	Standard Loading (Std. Estimate)
Sport Nutrition Knowledge	K1	1.000	-	-	-	0.825
Sport Nutrition Knowledge	K2	0.915	0.062	14.858	0.000	0.789
Sport Nutrition Knowledge	K3	0.941	0.063	14.849	0.000	0.788
Sport Nutrition Knowledge	K4	0.927	0.064	14.531	0.000	0.776
Sport Nutrition Knowledge	K5	0.990	0.062	15.851	0.000	0.827
Attitude	A1	1.000	-	-	-	0.821
Attitude	A2	0.913	0.071	12.940	0.000	0.752
Attitude	A3	0.939	0.070	13.434	0.000	0.778
Attitude	A4	0.862	0.071	12.080	0.000	0.708
Perceived behavioral control	C1	1.000	-	-	-	0.733
Perceived behavioral control	C2	1.110	0.092	12.103	0.000	0.793
Perceived behavioral control	C3	1.060	0.092	11.522	0.000	0.750
Perceived behavioral control	C4	1.028	0.088	11.649	0.000	0.759
Subjective norms	S1	1.000	-	-	-	0.776
Subjective norms	S2	0.900	0.076	11.781	0.000	0.711
Subjective norms	S3	0.925	0.077	11.983	0.000	0.723
Subjective norms	S4	0.981	0.077	12.698	0.000	0.762
Subjective norms	S5	1.004	0.079	12.633	0.000	0.759
Dietary behavior	B1	1.000	-	-	-	0.732
Dietary behavior	B2	0.957	0.091	10.565	0.000	0.683
Dietary behavior	B3	0.954	0.088	10.850	0.000	0.702
Dietary behavior	B4	1.124	0.093	12.093	0.000	0.790
Dietary behavior	B5	0.915	0.086	10.628	0.000	0.687

Note: Factor corresponds to the abbreviation of measurement item. For example, K1–K5 is the measurement item of sports nutrition knowledge, A1–A4 is the measurement item of attitude, C1–C4 is the measurement item of perceived behavior control, and S1–S5 is the measurement item of the subjective norm.

**Table 3:** Model AVE and CR results.

Factor	AVE	CR
Sport Nutrition Knowledge	0.642	0.900
Attitude	0.586	0.850
Perceived behavioral control	0.576	0.844
Subjective norms	0.557	0.863
Dietary behavior	0.518	0.843

**Table 4:** Discriminant validity: Pearson correlation and AVE root mean square (RMS) values.

	Sports Nutrition Knowledge	Attitude	Perceived Behavioral Control	Subjective Norms	Dietary Behavior
Sports nutrition knowledge	0.801				
Attitude	0.320	0.766			
Perceived behavioral control	0.364	0.389	0.759		
Subjective norms	0.355	0.388	0.401	0.747	
Dietary behavior	0.419	0.372	0.415	0.407	0.720

Notes: The blue numbers in the diagonal line are the AVE RMS values.

**Table 5:** Model fit indices.

Index	X^2^	df	*p*	X^2^/df	GFI	RMSEA	RMR	CFI	NFI	NNFI
Judgment standard	-	-	>0.05	<3	>0.9	<0.10	<0.05	>0.9	>0.9	>0.9
	257.729	220	0.041	1.171	0.929	0.025	0.030	0.988	0.923	0.986

Default Model: X^2^(253) = 3328.593, *p* = 1.000.

**Table 6:** Model regression coefficient.

X	→	Y	*SE*	*z* (CR)	*p*	Regression Coefficients
Sport Nutrition Knowledge	→	Attitude	0.062	5.908	0.000	0.401
Sport Nutrition Knowledge	→	Perceived behavioral control	0.053	6.395	0.000	0.451
Sport Nutrition Knowledge	→	Subjective norms	0.057	6.377	0.000	0.434
Sport Nutrition Knowledge	→	Dietary behavior	0.059	2.858	0.004	0.236
Attitude	→	Dietary behavior	0.054	2.742	0.006	0.187
Perceived behavioral control	→	Dietary behavior	0.069	3.203	0.001	0.231
Subjective norms	→	Dietary behavior	0.060	2.998	0.003	0.209
Sport Nutrition Knowledge	→	K2	0.062	14.903	0.000	0.790
Sport Nutrition Knowledge	→	K1	-	-	-	0.824
Sport Nutrition Knowledge	→	K5	0.063	15.739	0.000	0.823
Sport Nutrition Knowledge	→	K4	0.064	14.417	0.000	0.771
Sport Nutrition Knowledge	→	K3	0.063	14.809	0.000	0.787
Attitude	→	A4	0.073	11.964	0.000	0.710
Attitude	→	A3	0.072	13.170	0.000	0.776
Attitude	→	A2	0.072	12.847	0.000	0.758
Attitude	→	A1	-	-	-	0.816
Perceived behavioral control	→	C4	0.090	11.498	0.000	0.759
Perceived behavioral control	→	C3	0.094	11.337	0.000	0.747
Perceived behavioral control	→	C2	0.094	11.993	0.000	0.800
Perceived behavioral control	→	C1	-	-	-	0.728
Subjective norms	→	S5	0.079	12.630	0.000	0.757
Subjective norms	→	S4	0.076	12.652	0.000	0.758
Subjective norms	→	S3	0.076	11.965	0.000	0.720
Subjective norms	→	S2	0.076	11.832	0.000	0.713
Subjective norms	→	S1	-	-	-	0.782
Dietary behavior	→	B5	0.089	10.310	0.000	0.678
Dietary behavior	→	B4	0.096	11.719	0.000	0.783
Dietary behavior	→	B3	0.091	10.526	0.000	0.693
Dietary behavior	→	B2	0.093	10.250	0.000	0.674
Dietary behavior	→	B1	-	-	-	0.724

**Table 7:** Model fit indices.

Index	X^2^	df	*p*	X^2^/df	GFI	RMSEA	RMR	CFI	NFI	NNFI
Judgment standard	-	-	>0.05	<3	>0.9	<0.10	<0.05	>0.9	>0.9	>0.9
	322.694	223	0.000	1.447	0.909	0.040	0.066	0.968	0.903	0.963
**Index**	**TLI**	**AGFI**	**IFI**	**PGFI**	**PNFI**	**SRMR**	**RMSEA 90%CI**			
Judgment standard	>0.9	>0.9	>0.9	>0.9	>0.9	<0.1				
	0.963	0.887	0.968	0.734	0.796	0.086	0.030 ~ 0.049			

Default Model: X^2^(253) = 3328.593, *p* = 1.000.

## Data Availability

Requests for access to individual subject data may be made to Y.C.; please send an email to chenyao@hynu.edu.cn.
